# Immunotherapy in endometrial cancer: rationale, practice and perspectives

**DOI:** 10.1186/s40364-021-00301-z

**Published:** 2021-06-16

**Authors:** Wenyu Cao, Xinyue Ma, Jean Victoria Fischer, Chenggong Sun, Beihua Kong, Qing Zhang

**Affiliations:** 1 Department of Obstetrics and Gynecology, Qilu Hospital, Shandong University, 107 West Wenhua Road, Ji’nan, Shandong 250012 P.R. China; 2 Gynecology Oncology Key Laboratory, Qilu Hospital, Shandong University, Ji’nan, Shandong 250012 P.R. China; 3 Department of Pathology, Northwestern Medicine, Gynecologic Pathology Fellow, Chicago, Illinois USA

**Keywords:** Endometrial cancer, Immunotherapy, Biomarkers, Immune checkpoint blockades (ICBs), Vaccine, ACT, MetS, p53 mutant, Hormone aberrations

## Abstract

Tumor immunotherapy has attracted more and more attention nowadays, and multiple clinical trials have confirmed its effect in a variety of solid tumors. Immune checkpoint inhibitors (ICIs), cancer vaccines, adoptive cell transfer (ACT), and lymphocyte-promoting cytokines are the main immunotherapy methods. Endometrial cancer (EC) is one of the most frequent tumors in women and the prognosis of recurrent or metastatic EC is poor. Since molecular classification has been applied to EC, immunotherapy for different EC subtypes (especially POLE and MSI-H) has gradually attracted attention. In this review, we focus on the expression and molecular basis of the main biomarkers in the immunotherapy of EC firstly, as well as their clinical application significance and limitations. Blocking tumor immune checkpoints is one of the most effective strategies for cancer treatment in recent years, and has now become the focus in the field of tumor research and treatment. We summarized clinical date of planned and ongoing clinical trials and introduced other common immunotherapy methods in EC, such as cancer vaccine and ACT. Hormone aberrations, metabolic syndrome (MetS) and p53 mutant and that affect the immunotherapy of endometrial cancer will also be discussed in this review.

## Background

Uterine cancer, primarily EC, is the most common gynecologic tumor in developed countries [1] and ranks as the fourth highest among all cancers in number of estimated new cases among American females in 2020 [2]. The incidence rate of EC increased continuously (1.3% per year from 2007-2016) [3] partly due to the recent rise of nonendometrioid cases [4]. Most patients with EC are diagnosed at stage I because of notable early symptoms (irregular vaginal bleeding) [1]. Surgery including total hysterectomy and bilateral salpingo-oopherectomy with surgical staging is recommended as the standard management for medically operable patients, and adjuvant therapy (chemotherapy or radiotherapy) determinations are tailored on the basis of risk factors and pathologic findings, including age, tumor size and grade, lymphovascular invasion,lymph node involvement, degree of myometrium invasion, and lower uterine segment involvement. Among EC patients, 66.9% are diagnosed at local stage with a 5-year survival rate of 95.0%, conferring a good prognosis. However, 16% of EC patients have a metastatic disease with 5-year survival rate of 16.8%, which contributes disproportionally to disease mortality [5]. For those patients with uterine confined lesions and high-intermediate risk for recurrence, adjuvant radiotherapy (RT) is recommended. Carboplatin/paclitaxel systemic chemotherapy and RT can benefit those with high risk for recurrence. As for the patients with recurrent, extra-uterine lesions, systemic, combination therapy with or without RT is considered as suitable. However, effective treatment options are lacking for patients with advanced disease following standard therapy.

Immunotherapy has been recognized as a new, powerful approach for a variety of human carcinomas, with considerable clinical response seen in a portion of recurrent or refractory cases [6–8]. There are few second-line treatment options for endometrial cancer, and some progress has been made in immunotherapy in recent years. In March 2020, the national comprehensive cancer network (NCCN) recommended The Cancer Genome Atlas Research Network (TCGA) molecular typing of endometrial cancer for the first time and included it in the guidelines for diagnosis and treatment of endometrial cancer, which indicates the era of immunotherapy based on tumor microenvironment and genotype. With the continuous elucidation of the pathogenesis of endometrial cancer, more and more evidence shows that a large number of immune cells and cytokines can be seen in endometrial cancer tissue, and can stimulate endogenous anti-tumor immune response. Compared with other gynecological malignant tumors, endometrial cancer is most likely to benefit from immunotherapy [9–11]. The rationale for cancer immunotherapy can be summarized as reversing the tumors immuno-suppressive effects of the tumors or enhancing the inherent anti-tumor immune responses of the host [12]. The main cancer immunotherapy approaches include ICIs, cancer vaccines, ACT, and lymphocyte-promoting cytokines. Several methods such as oncolytic viruses and bispecific antibodies [13] are also emerging. Immunotherapy in EC, especially in those with advanced or metastatic disease, is drawing intense attention currently, as more than 50 clinical trials investigating various categories of immunotherapy in EC have been listed on the clinicaltrials.gov website (Table 1). For targeted therapy, the significance of biomarker's (Table 2) detection and clinical guidance is more clear. With the gradual application of immunotherapy in the clinic, more effort will be needed in this regard.

**Table 1:** Published or ongoing trials of immunotherapy in EC:

Mechanism of action	NCI identifie	Agents	Phase	Setting/cancer type	Patient number	Subtype contribution	Outcomes	Status
anti-PD-1 antibody	NCT01876511	Pembrolizumab	II	Advanced MMR-deficient Cancers including EC	Total:86, EC:15	all MMR-d	ORR, DCR, PR, SD, PD	published
	NCT02054806	Pembrolizumab	Ib	PD-L1 positive Advanced EC	24	POLE:1, MSI-high:1, MSS:17, Not evaluable:5	ORR, median PFS, median OS, PR, SD, PD	published
	NCT02628067	Pembrolizumab	II	Noncolorectal MMR-d cancers including EC	Total:233, EC:49	all MMR-d	CR, PR, ORR, median PFS, median OS, median DOR	published
	NCT02465060	Nivolumab	II	Noncolorectal MMR-d cancers including EC	Total:42, EC:13	all MMR-d	ORR, CR, PR, SD, PD, median PFS	published
Abti-PD-1 antibody+ Antiangiogenic therapy	NCT03015129	Pembrolizumab+ Lenvatinib	II	rEC	108	MSI:11,MSS:94	ORR, PFS, OS, DOR	published
anti-PD-L1 antibody	NCT01375842	Atezolizumab	Ia	rEC	15	MSI-H:1, MSS:7, unknown for MMR status:7	ORR, PFS,OS	published
	NCT02912572	Avelumab	II	rEC	33	MSS:16, MSI:17	OR, PFS6	published
	ACTRN12617000106336	Durvalumab	II	advanced EC	71	MSI:36, MSS:35	OTR, PFS, OS	published
anti-PD-L1 antibody+ anti-CTLA-4	NCT03015129	Durvalumab and Tremelimumab versus Durvalumab Monotherapy	II	rEC and Endometrial Carcinosarcoma	Total:56, EC:41	MSI:5, MSS:48, unknown:3	ORR, median DOR, PFS, PFS rate at 24 wks	published
tumor antigen loaded DC cancer vaccine	EudraCT 2009-016868-37	WT1mRNA-electroporated mature DCs (DCm-WT1- RNA)	I/II	Uterine Tumor	Total:6, serous EC:3	NA	PFS, OS, Oncologic outcome (CT Scan, CA125), Immune Response (WT1-specific T-cells, NK cells)	published
peptide cancer vaccine+ anti-tumor cytokine	NCT01580696	E39 peptide waccine+ GM-CSF	I/IIa	Ovarian and EC	Total:51, EC:9	NA	DFS	published
Chimeric peptide cancer vaccine	BB-IND-9803	B-cell epitopes of HER2-Th (MVF) epitope chimeric peptide vaccine	I	Metastatic and/or Recurrent Solid Tumors including EC	Total:24, EC:1	NA	antibody response, PR, SD	published
nucleic acid-based cancer vaccine+ anti-PD-1 antibody	NCT03313778	mRNA-4157 vaccine+ Pembrolizumab	I	Resectable/Unresectable Solid Tumors including EC	Total:33, EC:1	MSI	PR, SD, PD, neoantigen-specific T cell response	published
ACT+ hyperthermia+ anti-PD-1 antibody/ chemotherapy	NCT03757858	Autologous Adoptive T cell Transfer+ Hyperthermia+ Pembrolizumab/ Chemotherapy	I	Advanced Solid Tumors including EC	Total:33, EC:5	NA	ORR, DCR, Clinical Response (CR, PR, SD, PD)	published
anti-PD-1 antibody	NCT02628067	Pembrolizumab	II	Advanced Solid Tumors including EC	NA	NA	ORR	Recruiting
	NCT02630823	Pembrolizumab	I	Surgically Resectable EC	NA	NA	AEs	Active, not recruiting
	NCT02728830	Pembrolizumab	I	Gynecologic Cancers including EC	NA	NA	Fold change in tumor Immune Infiltrates	Active, not recruiting
	NCT02899793	Pembrolizumab	II	Advanced, Recurrent or Metastatic EC	NA	POLE,MSI	ORR,AEs	Recruiting
	NCT03241745	Nivolumab	II	Metastatic or Recurrent Uterine Cancer	NA	all dMMR(EC)	PFS	Recruiting
	NCT03568539	IBI308	Ib	Advanced or Metastatic Solid Tumors including EC	NA	NA	Overall Response Rate,CR,PR	Active, not recruiting
anti-PD-1 antibody+ Surgery	NCT03694834	Pembrolizumab+ Hysterectomy+ Surgical staging	I	Early Stage, High Grade Obesity-driven EC	NA	NA	TIL	Suspended
anti-PD-1 antibody+ Surgery+ Chemotherapy	NCT03932409	Pembrolizumab+ Vaginal Cuff Brachytherapy+ Paclitaxel+ Carboplatin	Ib	High Intermediate Risk EC	NA	NA	Proportion of patients completing three cycles	Not yet recruiting
anti-PD-1 antibody+ Chemotherapy	NCT02549209	Pembrolizumab+\Carboplatin+ Paclitaxel	II	Advanced, Recurrent or Metastatic EC	NA	NA	ORR	Recruiting
	NCT03276013	Pembrolizumab+ Doxorubicin	II	Advanced, Recurrent or Metastatic EC	NA	NA	PFS	Active, not recruiting
	NCT03981796	Dostarlimab+ Carboplatin- Paclitaxel	III	Advanced, Recurrent or Metastatic EC	NA	will do MMR evaluation	PFS	Recruiting
	NCT03914612	Pembrolizumab+ Paclitaxel+ Carboplatin	III	Stage III/IV or Recurrent EC	NA	will do MMR evaluation	PFS	Recruiting
anti-PD-1 antibody+Radiotherapy	NCT04214067	Pembrolizumab+ Radiation therapy	III	High Intermediate Risk Endometrioid EC	NA	all dMMR	3 year recurrence -free survival	Not yet recruiting
	NCT03955978	Dostarlimab+ Standard of Care Definitive Radiation	I	Inoperable EC	NA	NA	Safety and tolerability	Recruiting
anti-PD-1 antibody+PARP inhibitor	NCT03016338	Dostarlimab+ Niraparib	II	Advanced,Recurrent or Metastatic EC	NA	NA	Clinical benefit rate	Recruiting
	NCT03572478	Nivolumab+ Rucaparib	Ib/IIa	Advanced or Recurrent EC and Metastatic Castrate-Resistant Prostate Cancer	NA	NA	DLT rate	Recruiting
anti-PD-1 antibody+ Antiangiogenic therapy	NCT04197219	Pembrolizumab+ Axitinib	II	Advanced, Recurrent or Metastatic EC	NA	all dMMR	ORR,CR, PR	Not yet recruiting
	NCT02501096	Pembrolizumab+ Lenvatinib	Ib/II	metastatic Solid Tumors including EC	NA	NA	MTD,ORR,DLT	Active, not recruiting
	NCT03367741	Nivolumab+ Cabozantinib	II	Advanced, Recurrent or Metastatic EC	NA	NA	PFS	Suspended
	NCT03517449	Pembrolizumab+ Lenvatinib	III	Advanced, Recurrent or Metastatic EC	NA	will do MMR evaluation	PFS,OS	Active, not recruiting
	NCT03884101	Pembrolizumab+ Lenvatinib	III	Advanced, Recurrent or Metastatic EC	NA	will do MMR evaluation	PFS,OS	Recruiting
	NCT04157491	anti-PD-1 antibody+ Anlotinib	II	Advanced, Recurrent or Metastatic EC	NA	NA	ORR,CR, PR	Recruiting
anti-PD-1 antibody+IDO inhibitor	NCT02178722	Pembrolizumab+ Epacadostat (INCB024360)	I/II	Cancers including EC	NA	NA	TEAEs, ORR	Active, not recruiting
	NCT04106414	Nivolumab+ BMS-986205	II	Recurrent or Persistent EC or Endometrial Carcinosarcoma	NA	will do MMR evaluation	Best overall response rate	Recruiting
anti-PD-1 antibody+ anti-CTLA-4 antibody	NCT02982486	Nivolumab+ Ipilimumab	II	Nonresectable or Metastatic EC and Sarcoma	NA	all dMMR	CR,PR	Not yet recruiting
anti-PD-1 antibody+ 4-1BB antibody	NCT03917381	GEN1046	I/II	Malignant Solid Tumors including EC	NA	NA	DLT,AEs, Safety laboratory parameters	Recruiting
anti-PD-1 antibody+ CFTR inhibitor	NCT04014530	Pembrolizumab+ Ataluren	I/II	Metastatic EC and Colorectal Adenocarcinoma	NA	all dMMR(EC)	ORR	Recruiting
anti-PD-1 antibody+ Dual Adenosine Receptor (A2aR/A2bR) Antagonist	NCT03629756	Zimberelimab (AB122)+ AB928	I	Advanced Solid Tumors including EC	NA	NA	DLTs,AEs	Recruiting
anti-PD-1 antibody+ FRα ADC	NCT03835819	Pembrolizumab+ Mirvetuximab Soravtansine (IMGN853)	II	Advanced, Recurrent or Metastatic EC	NA	all MSS	ORR, PFS	Recruiting
anti-PD-1 antibody+ Hormone	NCT04046185	Toripalimab+ Progesterone	I	Early EC (preserve fertility)	NA	NA	Pathologic complete /patrial remission rate	Not yet recruiting
anti-PD-1 antibody+IAP	NCT04122625	Nivolumab+ Mimetic Debio 1143	Ib/II	Solid Tumors including EC, progressed during/immediately after anti-PD-1/PD-L1 treatment	NA	known MMR status	ORR	Recruiting
anti-PD-1 antibody+ Immune Modulatory Cocktail+ Radiotherapy+ Food supplement	NCT03192059	Pembrolizumab+ Immune Modulatory Cocktail (Vitamin D+ Lansoprazole Teva+ Cyclophosphamide+Aspirine,) +Radiotherapy +Curcumin	II	Refractory or Recurrent EC, CC, Uterine sarcoma	NA	NA	ORR	Recruiting
anti-PD-1 antibody+ multi-targeted RTK inhibitor	NCT03827837	Camrelizumab+ Famitinib	II	Advanced RCC/UC/CC/EC and Recurrent OC	NA	NA	Overall response rate	Recruiting
anti-PD-1 antibody+ PVRIG antibody	NCT03667716	COM701 monotherapy or COM701+ Nivolumab	Ia/b	Advanced or Metastatic Solid Tumors including EC	NA	NA	AEs ,DLTs,MTD, RDFE	Recruiting
anti-PD-L1 antibody	NCT02725489	Durvalumab+Vigil	II	Advanced Women's Cancers including EC	NA	NA	AEs	Active, not recruiting
	NCT03212404	Cosibelimab	I	Advanced or Metastatic Solid Tumors including EC	NA	NA	DLT,AEs,ORR	Recruiting
anti-PD-L1 antibody+ Chemotherapy	NCT02914470	Atezolizumab+ Carbplatin+ Cyclophophamide	Ib	Advanced Gynaecologic Cancer including EC,BC	NA	NA	Toxicity, Incidence of toxicity,AEs	Active, not recruiting
	NCT03503786	Avelumab+ Carboplatin+ Paclitaxel	II	Advanced, Recurrent or Metastatic EC	NA	NA	PFS	Not yet recruiting
	NCT03603184	Atezolizumab+ Paclitaxel+ Carboplatin	III	Advanced, Recurrent or Metastatic EC	NA	Will do MMR evaluation	OS,PFS	Recruiting
anti-PD-L1 antibody+ Antiangiogenic therapy	NCT03170960	Atezolizumab+ Cabozantinib	Ib	Advanced or Metastatic Solid Tumors including EC	NA	NA	MTD/RD,ORR	Recruiting
	NCT03526432	Atezolizumab+ Bevacizumab	II	Advanced, Recurrent or Metastatic EC	NA	will do MMR evaluation	Number of patients who experience CR or PR	Recruiting
anti-PD-L1 antibody+PARP inhibitor	NCT02912572	Avelumab/ Avelumab+ Talazoparib	II	Advanced, Recurrent or Metastatic EC	NA	POLE,MSI,MSS	PFS	Recruiting
	NCT03101280	Atezolizumab+ Rucaparib	Ib	Advanced Gynecologic Cancers inclunding EC, TNBC	NA	NA	AEs,DLTs,RP2D,Number of Dose Modifications due to AEs	Active, not recruiting
	NCT03951415	Durvalumab+ Olaparib	II	Advanced, Recurrent or Metastatic EC	NA	NA	PFS	Recruiting
anti-PD-L1 antibody+ Chemotherapy+ PARP inhibitor	NCT04269200	Durvalumab+ Carboplatin+ Paclitaxel+ Olaparib	III	Advanced, Recurrent or Metastatic EC	NA	will do MMR evaluation	PFS	Not yet recruiting
anti-PD-L1 antibody+ Antiangiogenic therapy+ PARP inhibitor	NCT03694262	Atezolizumab+ Bevacizumab+ Rucaparib	II	Advanced, Recurrent or Metastatic EC	NA	will do MMR evaluation	Overall Response Rate	Recruiting
anti-PD-L1 antibody+ IDO inhibitor	NCT02471846	Atezolizumab+ GDC-0919	Ib	Advanced or Metastatic Solid Tumors including EC	NA	NA	DLTs,AEs	Completed
anti-PD-L1 antibody+ anti-CTLA-4 antibody	NCT03015129	Durvalumab+ Tremelimumab	II	Persistent or Recurrent EC and Endometrial Carcinosarcoma	NA	NA	Overall Response Rate	Recruiting
anti-PD-L1 antibody+ anti-CTLA-4 antibody+ Radiotherapy	NCT03277482	Durvalumab+ Tremelimumab+ Radiotherapy	I	Recurrent Gynecologic Cancer including EC	NA	NA	MTD	Recruiting
anti-PD-L1 antibody+ anti-5T4 antibody-superantigen fusion protein	NCT03983954	Durvalumab+ Naptumomab	Ib/II	Advanced or Metastatic Solid Tumors including EC	NA	NA	AEs,MTD,RP2D	Recruiting
Dual Blockade of PD-1 and CTLA-4	NCT03517488	XmAb20717	I	Advanced Solid Tumors including EC	NA	all dMMR(EC)	AEs	Recruiting
anti-B7-H4 antibody	NCT03514121	FPA150	Ia/b	Advanced or Metastatic Solid Tumors including EC	NA	NA	MTD,RD,AEs	Active, not recruiting
anti-LAG-3 antibody	NCT03538028	INCAGN02385	I	Advanced or Metastatic Solid Tumors including EC	NA	all dMMR(EC)	TEAEs	Recruiting

*CR* Complete Response, *DCR* Disease Control Rate, *DOR* Duration of Response, *OR* Objective Response, *ORR* Objective Response Rate, *OS* Overall Survival, *OTR* Objective Tumour Response, *PD* Progressive Disease, *PFS* Progression-Free Survival, *PFS6* Progression-Free Survival at 6 months, *PR* Partial Response, *rEC* advanced or recurrent EC, *SD* Stable Disease, *ADC* Antibody-drug conjugates, *AEs* Adverse Events, *BC* Breast Cancer, *CC* Cervical Cancer, *DLTs* Dose-Limiting Toxicities, *MTD* Maximum Tolerated Dose, *OC* Ovarian Cancer, *RCC* Renal Cell Carcinoma, *RD* Recommended Dose, *RDFE* Recommended Dose for Expansion, *RP2D* Recommended Phase II Dose, *TEAEs* Treatment-Emergent Adverse Events, *TIL* Tumor Infiltrating Lymphocytes, *TNBC* Triple-Negative Breast Cancer, *UC* Urothelial Carcinoma.

**Table 2:** Biomarkers in ECs

Biomarkers	Expression (or association with) in ECs	Possible biomarker assessment
PD-L1	Over-expression	IHC
TMB	High	WES
TILs	Decreased	IHC
IDO1	Over-expression	ELISA
COX-2	Over-expression	IHC, WB
Glycodelin	Over-expression	ELISA

In 2013, TCGA [14] published an article regarding the integrated genomic classification of EC, classifying EC into four distinct molecular subtypes: Polymerase-ε(*POLE*) ultramutated, microsatellite instability hypermutated (MSI-H), copy-number low and copy-number high. The characteristics of these four subtypes which were determined through somatic mutations, microsatellite status and copy number alterations [14] are listed as follows:

*POLE* ultramutated ECs harbor DNA somatic mutations in the exonuclease domain within *POLE* gene and account for 7-12% of total EC patients [15, 16]. This subtype has a remarkably high mutation rate of 232 × 10–6 mutations/Mb. Patients of this subtype, though sometimes having high histological grades [17], usually have a good prognosis [16, 18–20], perhaps because of high immunogenicity [21] and chemosensitivity [22] due to DNA repair deficiency.

The MSI-hypermutated type of EC has diverse lengths of DNA microsatellites [14, 23, 24] in the genome and is also characterized by a high mutation rate (18×10-6mutations/Mb). Deficiency in the DNA mismatch repair (MMR) system, which is commonly due to gene mutations in *MLH1, MSH2, MSH6* or *PMS2*, is responsible for the phenotype of MSI EC. Somatic mutations of these four MMR-related genes, usually, *MLH1* promoter methylation [23], are often detected in MSI sporadic EC, whereas germ-line mutations of MMR-related genes are mainly found in hereditary EC, such as LS patients [25]. There is no statistically significant correlation between MSI status and clinical outcome [26, 27].

Both *POLE* and MSI subtypes of EC are generally considered as having high genome instability and immunogenic phenotypes [28, 29], with overexpressed immune-related biomarkers which will be further described in the following content.

Copy-number low ECs are also defined as microsatellite stability (MSS) ECs and have a low mutation rate (2.9 × 10–6 mutations/Mb). This type of EC accounts for nearly 60% of low-grade EC cases in TCGA, while only accounting for 8.7% of high grade ECs. Mutant *PTEN* and *PIK3CA* genes are found in 77% and 53% of this type of ECs respectively [14, 30]. Prognosis of the copy-number low subtype overlaps with MSI-H subtype with no obvious relationship between this subtype and outcomes [31].

The copy-number high subgroup is principally composed of serous and mixed histology tumors, with a residual portion of high grade endometrioid ECs. *TP53* mutation is generally detected (92%) in this type of EC, while *KRAS* and *PTEN* mutations infrequently occur. The prognosis of patients with this type of EC is poor and is worsened by unfavorable clinicopathological characteristics [24, 30–32]. Copy-number high/serous-like EC shares similar molecular patterns with high-grade serous ovarian carcinoma (HGSOC) and basal-like breast carcinoma, including highly mutant *TP53* gene (84%~96%), a low frequency of *PTEN* mutations (1%~2%) and similar focal somatic copy number alterations [14]. Both copy-number low and Copy-number high ECs are conventionally considered hypomutated ECs, characterized by low expression of immune-related biomarkers.

### Immune system in normal endometrium

The immune system in normal endometrium is mainly composed of endometrial epithelial cells, innate and adaptive immune cells, and inflammatory cytokines [33]. The endometrial epithelial cells, also a component of the mucosal immune system, have the functions of providing physical barriers [33], secreting defensins [34] or other immune mediators [35], and presenting antigens [36]. Both innate immune cells and adaptive immune cells participate in common surveillance and pathogen elimination in normal endometrium [33]. The inflammatory cytokines can recruit immune migratory cells [37] and modulate the immune network of the endometrium [33]. All these components’ functions are precisely regulated by sex hormones, such as estradiol and progesterone, and vary depending on the changes of menstrual cycles [33, 37, 38]. Macrophages and neutrophils are at the highest levels prior to menstruation [33] at which time they partly participate in the immunological protection and destruction of endometrial tissue [39]. Natural killer (NK) cells are inert during normal menstrual cycles and can mature to functional ones during pregnancy [40]. Regulatory T cells (Treg) can suppress ongoing immune responses in the endometrial microenvironment. T and B cells exist as aggregates in uterine mucosa [41–43], which are hormone-driven and absent in menopause [41]. The number of NK cells, Tregs, and T/B cell aggregates are increased during the menstrual cycle [33, 44], suggesting complicated immune modulation. Endometrial immunity, including all these components, plays a dual role in normal physiological processes by creating an immunosuppressive environment to avoid feto-maternal rejection while also protecting the disrupted endometrium from pathogens during menstruation [33].

### Rationale of Tumor-immunity interaction in EC

When the endometrium becomes cancerous, the immune microenvironment appears different from that of the normal endometrium. Endogenous or exogenous cancerogenic factors can directly modulate immune related signaling pathways or the host's defensive inflammation and also indirectly change the initial immune balance by tumor induced immunoediting. Endometrial immunity plays a paradoxical role during carcinogenesis, having both anti-tumor and tumor-promoting effects.

In the late 50’s, Burnet and Thomas introduced the “cancer immunosurveillance” hypothesis, which described that in immunocompetent hosts, tumor elicited a response from the immune system to evolve to control the malignant cell outgrowth [45, 46]. This conceptual model built a theoretical relationship between the immune system and tumor growth. However, controversy persisted with the results from several experimental studies [47, 48] providing little support for this hypothesis. The cancer immunosurveillance hypothesis was gradually abandoned. In 2002, Ikeda, Old and Schreiber [45, 49] first postulated a more complex “Cancer Immunoediting” model in which the immune system could both constrain and promote tumor development. It was defined by three distinct phases: elimination, equilibrium and escape. The tumor-immunity reciprocal activities in EC will be illustrated according to this classical model.

First, in the elimination phase, both innate and adaptive immune response are involved in the identification and cytotoxic elimination of EC cells [50]. Under stressful and dangerous conditions, the EC cells represent “altered self” phenotypes and express “non-self” antigens [51], which are phagocytized and processed by the dendritic cells (DCs) [52]. DCs are primed and then present these tumor-associated antigens to generate T cell responses including the production of CD8+ cytotoxic T cells (CTLs) and CD4+ T cells [52, 53]. CD8+ CTLs can directly kill EC cells while CD4+ T helper cells elicit a specific B cell response to provide both humoral and cytotoxic immune responses [54]. If the immune system completely wipes out the EC cells, the elimination phase can be termed as the endpoint of the cancer immunoediting process [55]. However, rare residual malignant cells may survive the elimination phase and enter the equilibrium phase. During the “Equilibrium” phase, the EC cells and immune system shape each other, and a temporal biological balance is established [56]. Several latent EC cells can reside in patients for decades and are maintained in a state of dormancy [57]. In this period, a complex interplay occurs between the elements of the immune system and EC, which will define the final outcome of the tumor’s existence. If the EC cells sculpt the immune system to produce an immunosuppressed environment, they will then escape the immunologic control and enter into the “Escape” phase [58]. The ECs resume growth and even forms distant metastases [55].

Understanding the mechanism underlying the transition of equilibrium phase to escape phase can help to develop immunotherapies. Researchers have now identified that the tumor cells secrete vascular endothelial growth factor, transforming growth factor-β, and indoleamine 2, 3-dioxygenase ( IDO) to inactivate or dampen immune cells [59–61]. Tumor cells can also escape immunosurveillance by losing the expression of tumor antigen and/or the major histocompatibility complex class I molecules [62] or through the immuno-inhibitory effects exerted by Treg and myeloid derived suppressor cells (MDSC). The up-regulation of immune checkpoints (Table 3), which block activated T cells through inhibitory pathways, is plays a crucial role in evading immune-surveillance [63]. In EC, when the immune system attempts to recognize and remove the tumor cells, two signals are necessary for enabling naïve T cell activation [64]. The first signal is the binding of T cell receptors (TCR) on T cells to antigenic peptide-bearing major histocompatibility complex on EC cells [55, 65], which alone is insufficient for T cell stimulation. The second signal is generated through the binding of costimulatory molecules, such as CD80 and CD86 (also known as B7-1 and B7-2), on the antigen-presenting cell (APC) to corresponding ligands (e.g. CD28) on the T cell [66]. This two-signal activation process can be negatively regulated by immune checkpoint pathways that can be exploited by EC cells to eliminate immune attack [67–69]. Programmed cell death 1 (PD1) and cytotoxic T lymphocyte antigen 4 (CTLA4) expressed on T or other immune cells and programmed cell death 1 ligand 1 (PDL1) expressed on EC cells are all principal components involved in immune checkpoint signaling. CTLA4 dampens costimulatory signaling of the CD28/B7 axis (the second signal) via competition with CD28 for binding to B7 ligands [69–71]. PD-1/PD-L1 engagement recruits tyrosine phosphatase SHP2 which dephosphorylates proximal signaling elements of T cell receptors signaling [72], thus producing a negative costimulatory effect and dampening T cell activation through T cell receptors signaling interference [69]. These immune checkpoint signals are defined as key targets for recently developed immunotherapies.

**Table 3:** Immune checkpoints in ECs

Immune checkpoint	Expression	ICBs on trials
PD-1	T-cell	Pembrolizumab nivolumab
PD-L1	Tumor cell	Atezolizumab Avelumab Durvalumab
CTLA-4	T-cell	Ipilimumab Tremelimumab
LAG-3	T-cell NK cell	INCAGN02385
B7-H4	tumor-associated macrophages Tumor cell	FPA150
TIM-3	T cells, Tregs, B cells, NK cells, DCs and macrophages	NA

### Biomarkers for EC immunotherapy

#### PD-L1

PD-L1 is one of several ligands for the PD-1 receptor. Tumor cells can up-regulate the expression of PD-L1 which binds to PD-1 on T cells activating the co-inhibitory signal in T cells and thus avoiding T cell cytolysis and facilitating tumor progression [73–75]. This pathway is one of the main targets of ICIs. Recent research has found that anti-PD-1/PD-L1 therapy has response rates of 36-100% in PD-L1-positive tumors compared with only response rates of 0–17% in PD-L1-negative tumors across all tumor types [75]. The expression of PD-L1 in the tumor microenvironment has been recognized as an important biomarker by demonstrating which patients are more likely to benefit from immunotherapy.

ECs show 75% expression of PD-1 and 25–100% expression of PD-L1, which are both the highest levels among gynecological cancers. However, PD-L1 expression patterns among different molecular subtypes of EC are controversial. In 2015, Howitt et al. [28] evaluated PD-L1 expression in 63 patients with EC. PD-L1 expression was more frequent in POLE and MSI tumors compared with MSS tumors in both intraepithelial immune cells based on presence vs total absence (P=0.02) and in peritumoral immune cells based on at least 10% expression (84% vs 56%, P=0.03), PD-L1 expression did not show significant difference in tumor cells among POLE, MSI, and MSS ECs. The higher expression levels of PD-L1 in POLE and MSI subtypes were also confirmed, respectively, by Howitt et al. and Pakish et al. [76]. Additionally, a study of 132 microsatellite stable, FIGO grade 2 endometrioid carcinoma patients [77] found that a subset of MSS ECs had higher expression of PD-L1. This study illustrated that among MSS tumors 48% (63/132) were PD-L1 positive and 16% (21/132) had more diffuse and/or especially strong PD-L1 expression. This PD-L1 over-expressed MSS cohort was associated with high numbers of tumor-associated CD3+ and CD8+ lymphocytes, sharing this characteristic with MSI ECs. In 2019, ESMO published a systematic review [78] in which the relationship of MSI status and PD-L1 expression was evaluated in several types of cancers. For EC, patients with both MSI and PD-L1 positive conditions only accounted for 3.1% of total ECs. Among all cancers studied, the cohort with MSI-high in combination with PD-L1 positive status was also a small percentage. Vanderwalde et al. [79] evaluated 11,348 cases (matched with 2189 cases) of 23 cancer types. The overall rate of PD-L1 positive cases was 25.4%, and only 26% of MSI-H cases were PD-L1 positive. Detecting the expression of PD-L1 by immunohistochemistry has been approved as a companion diagnostic test for the use of pembrolizumab in non-small-cell lung cancer (NSCLC), gastric/gastroesophageal junction adenocarcinoma, cervical cancer (CC), and urothelial cancer by the Food and Drug Administration (FDA) [80–84]. Furthermore, PD-L1’s predictive capacity has been seen across several other cancer types including head and neck and small-cell lung carcinoma [85–87]. Recently, for NSCLC patients with ≥50% PD-L1 expression on tumors, pembrolizumab was approved as a first-line therapy demonstrating a median overall survival (OS) of 30.2 months compared with 14.2 months for chemotherapy group. Based on these data, PD-L1 is a promising biomarker for immunotherapy in EC. However, research evaluating response to ICB based on the expression levels of PD-L1 has been inconsistent, and limitations still exist [88]. The heterogeneous expression of PD-L1 in the tumor microenvironment [88], lack of standard definition for positive-level of PD-L1 expression, and different detection methods resulting in lack of standardization across PD-L1 platforms are all problems which remain to be resolved.

#### Tumor mutational burden(TMB)

The second promising biomarker is TMB, defined as the number of mutations per coding region within the tumor genome [89]. In initial studies, mutation load was detected by whole exome sequencing comparing tumor DNA and matched normal DNA [90]. This expensive method is not widely applied in routine clinical care and now next-generation sequencing of targeted gene panels is commonly utilized to define TMB in clinical oncology [91, 92]. When normal cells undergo malignant transformation, cellular processes responsible for maintaining genomic integrity may be destroyed [93]. Cells are then unable to recognize or repair defects in DNA sequence or chromosome structure, resulting in accumulated mutations. Thus, it is no surprise that POLE-mutant and MSI ECs have a high TMB due to impaired DNA replication fidelity (POLE) and defective DNA MMR system (MSI-H) [14, 76]. DNA somatic mutations accumulate in cells, a fraction of which will further give rise to neoantigens [94]. These mutation-derived antigens can be displayed on HLA molecules on the surface of tumor cells [95, 96] and then be recognized by the immune system, especially after using T cell activating therapies [97–101]. Therefore, it is hypothesized that with higher TMB the ICB induced immune responses will be greater. Snyder et al. [102] first proposed TMB as a predictor of increased survival for patients who received ipilimumab or tremelimumab in melanoma. Subsequent trials defined TMB’s role across a wide variety of cancer types. For example, higher TMB was, found to benefit ICB therapies in NSCLC and small-cell lung cancer (SCLC) [103]. Currently, the FDA is considering approval of TMB-based assays as companion diagnostics for using ICB agents. As for MMR-d solid tumors which are defined as having a high TMB, recent work has demonstrated a high objective response rate (ORR) 53% to anti PD-1 therapy [104, 105]. These trials suggest the suitability of utilizing ICBs in TMB-high subtypes of ECs, which are mostly POLE and MSI ECs. Furthermore, although most cases of MSI-H solid tumors also have a high TMB, only 16% of TMB-high cases are MSI-H [91], suggesting that suitable cases for ICBs exist in other EC subtypes. What’s more, in addition to ICB therapy, TMB has shown predictive value in other immunotherapy modalities. Lauss et al. claimed that higher tumor mutation and neoantigen load could produce better clinical response (improved PFS and OS) in melanoma patients who were treated with adoptive T cell transfer therapy [106]. However, TMB also has some limitations as a biomarker. Some mutations in tumor cells can result in inactivation of the antigen presentation pathway causing no up-regulation in immune response [107, 108], impairing the predictive effects of TMB. Furthermore, there is no universal definition for high TMB [90]. Recent studies also evaluate the combination use of TMB with PD-L1 as biomarkers. Although the predictive roles of PD-L1 and TMB were shown to be independent, not correlated [109–111], and not co-associate in multiple trials [109], some trials did show a greater benefit of utilizing single anti-PD-1 and anti-PD-L1 therapy in patients with high TMB and PD-L1 expression [112]. Carbone et al. [109] showed that stage IV or recurrent NSCLC patients with both high tumor-mutation burden and ≥50% PD-L1 expression level had a better response to nivolumab than those with only one or neither of these biomarkers. The data suggest potential clinical value of combining TMB and PD-L1 as biomarkers for immunotherapy.

#### TIL

Another biomarker, tumor-infiltrating lymphocytes (TILs), was initially proposed by Clark at el. [113] to induce the lymphocytes in direct contact with tumor cells and/or infiltrating tumor nests. TIL includes a heterogeneous group of lymphocytes including effector T cells, tolerogenic or Treg cells, functionally exhausted T cells, NK cells, macrophages, DCs, MDSCs, and other immune cells [114]. These cell types have different competence in anti-tumor action. As the effector cells in TILs can elicit a cytotoxic antitumor immune response, the presence of TILs predicts better outcomes in several kinds of cancers such as melanoma [115], esophageal cancer [116], breast cancer (BC) [117] , colorectal cancer [118] and ovarian cancer (OC) [119, 120]. The predictive role of TILs was also demonstrated in EC by de Jong et al, who found high numbers of CTLs and high CD8+/ FOXP3+ ratio were associated with a longer disease free survival (DFS) while presence of CD45R0+ memory cells and high levels of CTLs predicted a greater OS [121]. As checkpoint inhibitors restore tumor cell recognition and T cell priming, pre-existing intratumoral CD8+T cells can exert anti-tumor response [122], suggesting the predictive role of TILs in immunotherapy. It has been found that patients with high “immunoscore”, representing the amount of infiltrating T cells, have better response to immunotherapies [123–125]. Because synthesized neoantigens due to accumulated DNA mutations are good binding sites for CD8+T cells, it makes sense that tumors with high TMB, such as POLE and MSI ECs, are the subtypes with higher TILs and benefit the most from immunotherapy [126, 127]. The TransPORTEC consortium performed assays on 116 high-risk ECs and confirmed that higher numbers of tumor-infiltrating T cells were found in POLE-mutant and MSI-H groups [128]. Recently, several other studies also showed increased TILs in POLE and MSI tumors compared with MSS tumors in EC and colorectal cancer [129, 130]. In 2017, Pakish et al. [76] compared the EC TME between the MSI-H group and MSS group (POLE-mutant or POLE-unknown cases were excluded) and reported increased immune cells, including granzyme B+ cells and activated cytotoxic T lymphocytes (CTLs, CD8+granzyme B+), were present in the stroma of MSI-H EC compared to MSS EC. Interestingly, this study further showed that Lynch syndrome (LS) related MSI-H ECs had reduced macrophages, increased CD8+ cells and activated CTLs compared with sporadic MSI-H ECs, suggesting the immune responses were affected by mechanisms underlying microsatellite instability.

#### Other emerging biomarkers

IDO1 is a type of tryptophan catabolic enzyme [131] which inactivates T cells and induces tumor immunotolerance [132]. IDO1 is over-expressed in a variety of tumor cells including EC cells [133, 134] and immune cells such as APCs, MDSCs and macrophages [132]. IDO1 expression (>25% ID O-positive) is more common in MMR-d EC (35%) than MMR-p EC (5%), specifically those with LS [135]. There is also a correlation between PD-L1 and IDO expression, as most PD-L1 positive tumors also express IDO [135], suggesting synergistic prediction in immunotherapy response.

Cyclooxygenase-2 (COX-2) is a rate-limiting enzyme responsible for the conversion of arachidonic acid to prostaglandins [136]. The production of COX-2 is usually at low levels in normal tissues [137] and is elevated in inflammatory states or cancer development [138, 139]. COX-2 is involved in a variety of procedures related to carcinogenesis, including angiogenesis, tumor cell proliferation, invasion, apoptosis inhibition, and immune evasion through prostaglandin E2 [140–144]. High levels of prostaglandin E2 can suppress interferon-γ(IFN-γ) production [145], antigen presentation [146] and IL-12 bio-synthesis and receptor expression [145, 147] and inhibit CTL proliferation and activation [148]. It has been reported that COX-2 overexpression was found in a series of cancers including CC, BC, gastric cancer, hepatocellular cancer and non-small cell lung cancer [149–151] and correlated with poor prognosis [152, 153]. In EC, a higher positive rate of COX-2 is related to more frequent cervical or extrauterine involvement (60.8%, p=0.02), higher grades (Grade 1 vs Grade 2 vs Grade 3: 13.6% vs 41.7% vs 60.9%, p=0.005), poor differentiation, and deep myometrial invasion [154, 155]. Additionally, COX-2 expression has been found to be associated with shorter DFS (p=0.09) [155] and disease relapse (p=0.03, univariate analysis) [156]. As for immune evasion in EC, COX-2 expression is inversely correlated with the degrees of CD8+T cell infiltration [156, 157]. This suggests that COX-2 can be used as a potential biomarker for immunotherapy.

Glycodelin, also termed as PAEP, is a secreted glycoprotein isolated from endometrium, decidua, seminal plasma and amniotic fluid [158]. It plays a suppressive role in cancer immune response which is similar to its function in maternal immune tolerance [159, 160]. The immuno-modulatory effects of glycodelin involve a variety of immune cells including DCs, NK, macrophages, T and B cells [161]. Over-expression of glycodelin is thought to play a role in carcinogenesis including the promotion of angiogenesis, cell proliferation, differentiation, and invasion [161], and is found in multiple gynecological cancers, such as EC and OC [162]. The expression of the immunosuppressive isoform of Glycodelin, termed Glycodelin A, is related to poor outcomes (p=0.003) and is an independent predictor for patient survival (p=0.002) in EC [163]. Although it has been identified as an important biomarker for immuno-modulatory functions in a certain cancers [164, 165], there is no data about its practical use in predicting immunotherapy efficacy, which deserves further study.

Several immune-related genetic signatures should be further investigated as alternative biomarkers for immunotherapy in EC. The AT-rich interaction domain 1A (*ARID1A*) mutation has been reported to not only facilitate ICB therapy but also potentially predict the efficacy of ICB therapy [166, 167]. Deficient *ARID1A* results in decreased MMR protein [168], higher mutation frequency [169] and increased TIL [170] and PD-L1 expression [171]. In EC, *ARID1A* alteration was correlated with higher infiltration of six common immune cells including CD8+ T and CD4+ T cells, B cells, neutrophils, macrophages and DCs [167], supporting the view that deficient ARID1A might be a potential predictor for ICB efficacy in EC. For hypomutated EC types including CN-low and CN-high/serous-like ECs, recent studies noted the correlations of several gene mutations with low neoantigen load, such as *CTNNB1* alteration in CN-low EC and *MYC* amplification and *PIK3CA* alteration in CN-high EC [14, 172]. These genetic biomarkers might predict poor effects of immunotherapy response and can be used to select potential candidates with non-immunogenic types of EC for immunotherapy [173].

### ICBs

ICBs are a group of monoclonal antibodies targeting immune checkpoint proteins which mainly transmit co-inhibitory signals during T cell activation [174]. In the immune microenvironment of EC, tumor elicited immunosuppression is mainly generated from the conjugation of over-expressed PD-L1 and PD-L2 on EC cells to PD-1 receptors on tumor infiltrating CD4+/ CD8+ T cells and CTLA-4 expressed on Treg to B7-1/B7-2 (the ligand of stimulatory receptor CD28) expressed on APC. ICBs can decrease the negative immunomodulation exerted by tumor cells/Tregs through PD-1/PD-L1 and CTLA-4/B7-1, B7-2 pathways and thus restore antitumor effects of T cells [67, 175, 176]. Since 2011 when ipilimumab (anti-CTLA4) was first approved by the FDA for patients with metastatic melanoma, various ICBs, such as nivolumab, pembrolizumab, atezolizumab, avelumab and durvalumab, have continuously been approved by the FDA for treating a wide range of malignancies, with approval likely for additional tumor types in the near future. POLE and MSI ECs are favorable candidates for ICBs therapy because of high expression of immune-related biomarkers which have been previously illustrated. One of the ICBs, pembrolizumab (anti-PD-1 agent), has been approved by the FDA for patients with MSI or MMR-deficient solid tumors which are resistant to conventional treatment, supporting ICB therapy in the treatment of advanced MSI EC. Additionally, the expression of canonical biomarkers and novel evidence of ICB adaptability have been detected in a portion of EC patients aside from POLE/MSI types, such as the MSS EC.

#### ICBs in POLE/MSI EC

Pembrolizumab, a PD1 blockade, is the first ICBs whose clinical activity has been investigated in EC. A phase II trial (NCT01876511) by Le et al. [104] evaluated the efficacy of monotherapy pembrolizumab (10 mg/kg every 14 days) in patients with advanced MMR-deficient cancers (12 tumor types). This trial included 15 MSI EC patients. For the EC cohort, after trial completion, the disease control rate was 73% including 3 (20%) patients with complete response (CR), 5 (33%) patients with partial response (PR) and 3 (20%) patients with stable disease (SD). The ORR of the EC cohort was 53%. Treatment related adverse events (AEs) were observed in 74% of patients, most of which were at low grade and manageable, such as endocrine disorders (i.e. hypothyroidism). This trial expanded the therapeutic value of ICBs from canonical MMR-d colorectal cancers to more MMR-d tumor types and drew researchers’ attention to apply ICBs in MSI EC.

Pembrolizumab was then evaluated in a multicohort phase Ib KEYNOTE-028 (NCT02054806) study which enrolled patients with PD-L1 positive advanced solid tumors [177]. Twenty-four advanced PD-L1 positive EC patients were eligible, among whom, only 2 patients were defined as POLE and MSI-high respectively while others were non-MSI-high or not evaluable. Twenty-three EC patients were included in the final efficacy analysis, the ORR was 13.0% (95% CI, 2.8% to 33.6%), the median PFS at the data cutoff was 1.8 months (95% CI, 1.6 to 2.7 months), and the median OS was not reached (95% CI, 4.3 months to not reached). PR, SD, and progressive disease (PD) were observed respectively in 3 (13.0%), 3 (13.0%), and 13 (56.5%) patients. The therapy in the single POLE patient was particularly effective as he achieved PR,while the MSI-high patient had PD. Grade 3 treatment-related AEs only occurred in four patients and no grade 4 AEs or AE induced treatment discontinuations were observed. This study suggested a superior benefit of PD-1 blockade in POLE ECs, but the analysis was limited by small cohort sizes.

In 2019 a phase II KEYNOTE-158 study (NCT02628067) evaluating pembrolizumab in MMR-d noncolorectal carcinoma was published. This study enrolled a larger cohort of EC patients [178]. Forty-nine patients with progressive MSI EC, intolerant to standard therapy, were eligible. The median follow up time was 13.4 months. The total ORR for all 23 MMR-d tumor types was 34.3% (95% CI, 28.3% to 40.8%). For EC cohort, 8 (16%) patients achieved CR, 20 (41%) patients achieved PR, the ORR was 57.1% (95% CI, 42.2 to 71.2) , the median PFS was 25.7 (95% CI, 4.9 to NR), and the median OS was not reached. Specifically, EC was one of the tumor types with the most frequent CR in this study. Furthermore, 37 EC patients had a ≥30% reduction in tumor size among 47 EC patients with a tumor size change from the baseline data, and EC was the most common tumor with size reduction in the study. Severe AEs occurred at a low rate as only 3 patients experienced grade 4 treatment-related AEs and one patient died of treatment induced pneumonia were reported. These data show the impressive value of using pembrolizumab in MSI EC. Ongoing trials are investigating combination therapy of pembrolizumab with other therapeutic methods in MSI EC. A phase I/II study (NCT04014530) of MSI metastatic EC and colorectal adenocarcinoma are recruiting volunteers for pembrolizumab plus ataluren combination therapy with the primary outcome measure of ORR. Trials investigating combination of pembrolizumab with radiotherapy (NCT04214067) or anti-angiogenic agent (NCT04197219) in MSI EC have also been registered but not yet recruited yet.

Recently, nivolumab, a new PD-1 blockade, has shown great activity in MMR-d colon cancer. Mounting preclinical or clinical studies are ongoing to broaden its usage in more MMR-d tumors. In 2020, a newly published study noted that the effect of nivolumab was evaluated in a phase II study (NCT02465060) which enrolled 42 relapsed MMR-d non-colorectal cancer patients [179]. Thirteen refractory/relapsed MSI EC patients were eligible. Nivolumab was administrated intravenously 3 mg/kg every 2 weeks (28-day cycles) and 480 mg every week after cycle 4. The total ORR was 36% (15/42; 90% CI,23.5% to 49.5%). Three patients (7%) achieved CRs, 13 patients (29%) achieved PR and 9 patients (21%) achieved SD. As for the EC cohort, 2 patients had CR (2/13, 15%), 3 patients had PR (3/13, 53%) and 7 patients showed decreased tumor size from baseline (7/13,53%). Treatment-related toxicities were mild and at low grades (most were grade 1-3), with only 2 grade 4 AEs in 3 patients and no grade 5 AEs. This trial noted a promising effect of nivolumab on treating MSI EC. An ongoing phase 2 study (NCT03241745) of nivolumab monotherapy is recruiting patients with MSI metastatic /recurrent uterine cancer, which will further provide evidence on using nivolumab in MSI EC.

Blocking PD-L1 is another significant mechanism for ICBs in EC. In 2017, the effect of a PD-L1 blockade, atezolizumab, was initially evaluated by a phase Ia study (NCT01375842) in 15 advanced or recurrent endometrial cancer (rEC) patients [180]. Among them, 1 was MSI-H, 7 were MSS, and 7 had unknown MMR status. Atezolizumab 1200 mg or 15 mg/kg intravenous every 3 weeks was administered to the patients. PD-L1 expression was evaluated in the tumor samples with expression on immune cells (IC) comprising ≥ 5% of the tumor defined as PD-L1 positive (IC2/3). After treatment, the ORR was 13% (2/15) in total and 40% (2/5) in IC2/3 patients. As for the 2 responders who achieved PR, one was MSI-H and had moderate TILs infiltration (IC2,10%TILs), the other was MSS but had heavy TILs infiltration (IC3,70%TILs) which was similar to the tumor immune micro-environment in MSI ECs. Duration of objective response (DOR) for these 2 cases were 7.3 and 8.1+ months, respectively. Median PFS was 1.7 months (range, 0.6-11+); median OS was 9.6 months (range, 0.6-11.8+). Atezolizumab showed a relatively safe profile in rEC. Among 7 (47%) patients with any treatment related AEs, 5 patients had G1-2 AEs, no G4-5 AEs was observed, and only 2 patients had SAEs including colitis and rash. This trial noted that MSI status, high TILs, and PD-L1 positivity may be factors for atezolizumab therapy.

Aside for atezolizumab, avelumab is also a promising immune check point inhibitor targeting PD-L1 in EC. Konstantinopoulos et al. [181] performed a phase II study (NCT02912572) of avelumab in patients with recurrent/persistent EC. Thirty-three patients were enrolled in this study and were divided into 2 cohorts: MMR-d (17/33) and MMR-proficient (16/33), with no POLE patients enrolled. Avelumab 10 mg/kg was administered intravenously every 2 weeks until tumor progression or unacceptable toxicity. Two MMR-d patients were not enrolled in protocol treatment and were excluded from terminal analyses. At the first stage, only 1 patient from the MMR-p cohort achieved both objective response (OR) and PFS at 6 months (PFS6), so this cohort was closed because of futility and did not enter into next stage, the ORR was 6.25% (95% CI 0.16% to 30.2%). The MMR-d cohort completed 2 stages of treatment. In this cohort, the ORR was 26.7% (95%CI, 7.8% to 55.1%), 4 ORs including 1 CR and 3 PRs were observed. Six patients exhibited PFS6 (40.0%; 95% CI 16.3% to 66.7%, including all 4 ORs), 4 of whom were still receiving the treatment at the data cutoff. Treatment-related toxicity was tolerable. All treatment-related AEs were G1-3, with no G4 or G5 AEs occurring in either cohort. This study suggested promising effect of avelumab in MMR-d EC.

Durvalumab, another PD-L1 inhibitor, also showed impressive effects in MSI ECs. In 2019, 71 patients with advanced EC were enrolled in a phase II PHAEDRA trial (ANZGOG1601) [182] to evaluate the effect of durvalumab therapy. These patients had all experienced progression after 0-3 lines of chemotherapy prior to the durvalumab treatment. Thirty-six patients were defined as MMR-d and 35 patients were MMR-p. Durvalumab was administrated 1500mg intravenous Q4W. The objective tumor response (OTR, including CR or PR by Response Evaluation Criteria In Solid Tumors) rate for MMR-d cohort was 47% (17/36, 95% CI 32-63%), including 6 CR and 11 PR, median PFS was 5.5, 12-month OS was 71% and median OS was not reached. In contrast,for the MMR-p cohort, the OTR rate was only 3% (1/35,95% CI 1-15%), 1 PR and 10 SD were observed, median PFS was 1.8 months, 12-month OS was 51%, and median OS was 11.5 months.

As for CTLA-4 targeted therapy, data are limited in POLE/MSI EC. Ipilimumab and tremelimumab are two significant anti-CTLA-4 monoclonal antibodies in the clinical studies of melanoma, mesothelioma, NSCLC [183–185] and other tumors. Several trials of CTLA-4 combination therapy in EC are ongoing to identify the possible clinical efficacy. Rubinstein et al. [186] reported an interim analysis of a phase II trial (NCT03015129) comparing the combination of durvalumab and tremelimumab (DT arm) to durvalumab monotherapy (D arm) in patients with advanced EC and endometrial carcinosarcoma. Fifty-six patients were enrolled and divided equally into D arm and DT arm, 5 (9%) patients were MSI-H, 48 (86%) patients were MSS and 3 (5%) patients had unknown MMR status. Twenty-seven patients per arm were enrolled in the evaluation and a modest clinical activity was observed. 40% (2/5) of MSI patients had ORs (D arm: 1 CR, DT arm: 1 PR). The efficacy appeared poorer in MSS patients with only 5 (10.4%) achieving ORs (D arm: 1 CR,2 PR, DT arm: 1 CR, 1 PR). In this trial, G3 and G4 treatment-related AEs were observed in 11 (DT arm: 9, D arm: 2) and 4 (DT arm: 3, D arm: 1) patients, respectively. However, serious AE induced by this kind of combination strategy was described in a case report by Mahmood et al [187]. They reported on a 75-year-old Caucasian female with advanced EC received durvalumab 1500 mg plus tremelimumab 75 mg as a combination therapy and only had grade 1 skin pruritus at the first cycle of treatment. However, after 4 weeks, she developed a fulminant immune-mediated myocarditis and was treated with high-dose intravenous steroid. She did experience cardiac function recovery but had progression of metastatic lesions. Recently, a phase II study (NCT02982486) began investigating the combination of ipilimumab (an anti-CTLA-4 agent) and nivolumab in patients with nonresectable/metastatic sarcoma or EC with somatic MMR deficiency. This trial will evaluate the efficacy of ipilimumab 1 mg/kg every 6 weeks plus nivolumab 240 mg every 2 weeks in an estimated 60 participants, and CR and PR will be the primary outcome measures.

#### ICBs in MSS EC

Although POLE/MSI ECs have shown considerable sensitivity to ICB therapy, these two subtypes only comprise a minority portion of EC cases. Most of the endometrioid (72%) and serous (98%) EC patients fall into the copy number-low or copy number-high subtypes, with no MSI characteristics [188]. Sporadic responses of non-POLE/MSI ECs were seen in the trial results listed in the prior paragraphs. Furthermore, Goodman et al. [189] claimed that patients with MSS tumors but marked as TMB-high might benefit from immunotherapy, suggesting complicated factors contributing to clinical outcomes aside from the known biomarkers.

In March 2019, Makker and his colleagues [190] published an interim analysis of a phase 2 trial (NCT03015129) which assessed the combination of pembrolizumab and lenvatinib in advanced endometrial cancer. Eligible patients were unselected for microsatellite instability or PD-L1 expression status. At the interim analysis cutoff, 53 patients were included in this interim evaluation, 85% (45/53) of whom were defined as MSS and 25% were PD-L1-positive. At 24 weeks, 39.6% (21/53) of patients had an OR. The median follow-up for PFS was 7.7 months. Notably, objective responses and tumor size reduction from baseline were respectively observed in 35.6% (16/45) and 80% (36/45) of MSS patients. This was higher than what was reported in former studies of advanced EC [191, 192]. This result led to the approval of pembrolizumab plus lenvatinib by the FDA for the treatment of advanced EC, a significant breakthrough. In October 2019, the final result of the advanced EC cohort in this trial was published. At data cutoff, 108 patients were included in the final analysis, MSI vs MSS was 13% (11/108) vs 87% (94/108), 38% (41/108) of total patients reached ORR at 24 weeks (95% CI, 28.8-47.8%), the median duration of response was 21.2 (7.6-NR) months. The ORR at 24 weeks was 36.2% (34/94) vs 63.6% (7/11) for MSS vs MSI, 7 (7.4%) MSS patients and 1 (9.1%) MSI patient achieved CR, and 28 (29.8%) MSS patients and 6 (54.5%) MSI patients achieved PR. The median duration of response was not reached for the MSS cohort and was 21.2 months for the MSI cohort. In conclusion, the authors reported promising activity of pembrolizumab plus lenvatinib in advanced EC regardless of MSI/MMR status. Two phase 3 studies (NCT03517449, NCT03884101) are ongoing to further examine lenvatinib plus pembrolizumab versus chemotherapy regimens of doxorubicin/paclitaxel/carboplatin in advanced EC with known MMR status, in order to provide more data to guide the use of this therapy.

There are also some case reports discussing the latent benefit of using ICBs in MSS EC. For example, in 2019, Oh and Chae [193] reported a 57-year-old, MMR-proficient EC patient with diagnosis of stage IV endometrial adenocarcinoma and PD-L1 negative status. After completion of neoadjuvant chemotherapy, total abdominal hysterectomy and bilateral salpingo-oophorectomy with optimal surgical debulking and 3 cycles of chemotherapy, she had PD at 4 months and wanted to avoid continued chemotherapy. She was then introduced to a combination PD-1 and CTLA-4 blockade, which included nivolumab 3 mg/kg every 2 weeks and ipilimumab 1 mg/kg every 6 weeks. Fortunately, she had a deep and durable response after this treatment with 79% shrinkage of tumor size in 1-year, continued reduction of metastatic lesions, and PR noted by cross-sectional imaging. This case report highlights a satisfactory clinical response to ICB in an MSS EC patient who would typically have been considered an unfavorable candidate for immunotherapy.

Although clinical responses are sometimes seen in MSS EC, conclusive biomarkers recognizing responders are still lacking [188]. Current studies on treating MSS EC by immunotherapy mainly concentrate on combining ICBs with other agents to get a higher anti-tumor effect as described before. There exists a series of ongoing trials studying the combination of ICBs with chemotherapy, radiotherapy, poly (ADP-ribose) polymerase (PARP) inhibitors, antiangiogenic drugs, and other target agents. Patients enrolled in these assays will be evaluated for MMR. The prospective outcomes of patients with diverse MMR status in those trials will enlighten us on the subject of immunotherapy in MSS EC patients. Representative trials are listed as follows.

##### ICBs & Antibody-drug conjugates(ADCs)

ADCs are a group of agents composed of a specific antibody targeting tumor-associated antigen conjugated with a cytotoxic effector compound [194, 195], which have been identified as effective therapies in solid tumors [196, 197]. Mirvetuximab soravtansine (IMGN853), as an ADC, involves a humanized anti-FRα monoclonal antibody targeting selective tumor cells and tubulin-disrupting maytansinoid DM4 as a cytotoxic module [195, 198]. After the binding of IMGN853 to FRα on tumor cells, the drug is internalized, leading to an accumulated intracellular concentration of DM4 [199]. This produces an antimitotic effects, and the tumor cells are killed [200, 201]. The combination of IMGN853 with pembrolizumab is being investigated in patients with MSS recurrent or persistent EC in an ongoing phase 2 study (NCT03835819). Thirty-five participants are estimated to be enrolled and will receive both pembrolizumab and IMGN853 administered intravenously once every 3 weeks. The primary outcome measures include ORR and PFS.

##### ICB & antiangiogenic agents

Aside from the previously described trials of pembrolizumab plus lenvatinib, several ongoing trials are researching the combination therapy of ICBs with antiangiogenic agents. There is a phase II, single arm study (NCT03526432) investigating atezolizumab plus bevacizumab which is now recruiting patients with advanced EC. Patients’ MMR status will be determined before they enter into the trial. PR and CR will be measured for the evaluation of clinical efficacy as well as PFS, OS, and number of patients experiencing toxicity and immune related response.

##### ICB & PARPi

A multicenter, placebo-controlled, phase III study (NCT04269200) is ongoing to investigate the combination therapy of first-line chemotherapy, durvalumab, and PARPi in patients with newly diagnosed advanced EC. Six-hundred-ninety-nine EC patients with known MMR status are estimated to be enrolled in this trial. This study includes 3 arms: patients in arm A receive standard chemotherapy of carboplatin and paclitaxel and placebos as the control group, patients in arm B receive chemotherapy plus durvalumab, and the residual patients in arm C receive the combination therapy of chemotherapy, durvalumab, and olaparib. The primary outcome measure is PFS. Since *ARID1A* gene deficiency in some EC patients is related to DNA homologous recombination repair deficiency [202], PARPi may be effective in these *ARID1A* mutated patients, which is supported by a series of preclinical trials [203, 204].

##### ICBs & IDO1 inhibitors

BMS-986205(69,(R)-N-(4-chlorophenyl)-2-((1S,4S)-4-(6-fluoroquinolin-4-yl)cyclohexyl)propanamide) is an IDO1 inhibitor with potential [132] therapeutic benefit, as it has been demonstrated that IDO1 is partly responsible for the formation of resistance to ICBs [131]. Combination of IDO1 inhibitor (such as BMS-986205) with ICBs might be an alternative strategy for EC patients. The combination of nivolumab with BMS-986205 in recurrent or persistent EC and endometrial carcinosarcoma is being investigated in an ongoing phase 2 trial (NCT04106414). Enrolled patients will be divided into a nivolumab monotherapy (480 mg every 4 weeks) group and a nivolumab (480 mg every 4 weeks) plus BMS-986205 (100 mg every 4 weeks) group. MSI/MSS evaluation must be done prior to entering into the trial. The best overall response rate determined by RECIST 1.1 is the primary outcome measure.

#### Emerging immune checkpoints and ICBs

##### Lymphocyte-activation gene 3 (LAG-3, CD233)

New ICBs are being researched to provide a large selection of therapeutic drugs. LAG-3 is a key immune inhibitory receptor mainly expressed on activated T and NK cells [205]. Previous studies identified the major histocompatibility complex class II (MHC-II) as the major ligand of LAG-3 [206, 207]. The combination of LAG-3 with MHC-II can inhibit the activation of CD4 + helper T cells which depend on MHC-II/CD4 interaction [208]. However, this mechanism fails to explain the functional suppression of CD8+ T cells and NK cells by LAG-3 [209] or the T cell activation by several anti-LAG3 mAbs which fail to target LAG-3/MHC-II signaling [210–213]. In January 2020, Wang et al. [213] suggested fibrinogen-like protein 1, which is highly produced by cancer cells, as the major ligand for LAG-3. The fibrinogen-like protein 1/LAG3 combination could induce LAG3-dependent T cell suppression and result in tumor immune evasion. These findings support LAG-3 as an important target for ICB development. A phase 1 study (NCT03538028) of INCAGN02385 (an antagonist antibody targeting LAG-3) in patients with advanced tumors including MSI EC is now in the recruiting period, and the primary outcome measured will be the number of treatment-emergent adverse events (TEAEs).

##### B7-H4

B7-H4 is a coinhibitory molecule contributing to the B7 family [214, 215]. It binds to unknown receptors on activated T cells [216] and transmits negative immuno-modulatory signals, thus promoting immune escape through negative regulations of cytokine secretion, cell proliferation, and the T cell cycle [217]. B7-H4 is highly expressed on tumor-associated macrophages [218] and in many solid tumors, such as OC, BC, lung cancer, renal cell cancer, and pancreatic cancer [219–224]. Different results were observed in studies testing the expression levels of B7-H4 in EC. Miyatake et al. [225] found that with progression of the endometrial mucosa from a normal phenotype to hyperplasia and malignancy, a higher proportion and intensity of B7-H4 expression was observed on the surface of the cells. Additionally, high risk ECs had a higher intensity and proportion of B7-H4 positivity compared with low risk ones ( P=0.001 and P=0.032, respectively), and B7-H4 was positively associated with CD3+ and CD8+ TILs ( P=0.039 and P=0.031, respectively). However, Vanderstraeten et al. [134] and Liu et al. [226] found a high rate of B7-H4 positivity (90-100%) in EC tissues regardless of the cancer settings (primary or metastatic) and the normal endometrial samples in these two studies were all (100%) B7-H4 positive. Also, Bregar et al [227] claimed that the expression levels of B7-H4 were similar among ECs with different microsatellite status, grades, histology determinations, and immune cell infiltrations. Recently, the role of B7-H4 in tumor development has been identified. It is tightly associated with tumor aggressiveness and metastasis by functioning to promote cancer cell proliferation, invasion, and anti-apoptosis [217]. Therefore, B7-H4 has been considered a novel target for immunotherapy in cancer. An ongoing (NCT03514121) phase Ia/b trial is now testing FPA150 (an anti-B7-H4 antibody) in patients with advanced or metastatic solid tumors including EC. This study will evaluate the maximum tolerated dose (MTD) and/or recommended dose (RD), and AEs will be recorded.

##### T cell immunoglobulin and mucin-domain containing-3 (TIM-3)

TIM-3 is another intriguing immune checkpoint molecule which is similar to PD-1 or CTLA-4 [228]. TIM-3 is not only expressed on effector T cells (CD4+ and CD8+ T cells) but is also found on Tregs, B cells, NK cells, and antigen-presenting cells including DCs and macrophages [229]. TIM-3 can induce the exhaustion of cytotoxic T cells and activation of Tregs resulting in immune-tolerance via a mechanism separate from the PD-1/PD-L1 axis [230–234]. The expression of TIM-3 has been identified in a variety of solid tumors, such as cervical, urothelial, gastric, and prostatic carcinomas and melanomas [234–238]. As for EC, Moore et al. [239] demonstrated that focal expression of TIM-3 was found in all EC cases, but stronger expression was observed in MMR-d cases (66% of MMR-d vs 12% of MMR-p, P=0.00002) with particularly intense TIM-3 staining in *MLH1*-hypermethylated and *MSH6* loss cases. Furthermore, intermediate and high-grade EC are more likely to express TIM-3 compared with low grade tumors (P=0.02). Although this immune checkpoint has shown potential value as a target for ICB therapy in EC, no related trials or research exist, so the clinical usage of TIM-3 in EC remains to be explored.

### Cancer vaccine

A cancer vaccine is a form of active-specific immunotherapy (ASI) [240] which harnesses the host’s immune system to attack the tumor cells [241]. The rationale for designing cancer vaccines is similar to that of vaccines against infectious diseases [240], which can be summarized as employing disease/tumor associated specific antigens to elicit APC mediated CD8+ and CD4+ T cell responses [242] and induce a persistent immune memory [243]. Under the tumor elicited immuno-tolerant environment [244], high levels of cancer vaccine are necessary for the expansion of DCs [245], which in turn promote the anti-tumor effects of T cells. Cancer vaccines are further classified as prophylactic vaccines and therapeutic vaccines [246]. The prophylactic vaccines, such as the HPV and HBV vaccines, block the infection of tumor-driving viruses and are only preventive if its administration precede tumor occurrence [246]. There is not the main topic discussed here. The categories of therapeutic cancer vaccines mainly include tumor/immune cell vaccines, peptide vaccines, and genetic vaccines comprised of DNA, RNA, and viral vaccines [247].

### Peptide vaccine

Specific tumor associated antigens over-expressed on EC cells can be artificially processed to generate peptide cancer vaccines. One example of such is the WT1 peptide vaccine. The wild-type *WT1* gene has been characterized as an oncogene in many malignancies [248] and is specifically highly expressed in various gynecological cancers [249, 250]. The product of the *WT1* gene is a tumor associated antigen, which can be recognized by T cells when associated with MHC class 1 molecules. Since 2004, a number of preclinical studies and case reports of WT1 immunotherapy have been published [251, 252]. Direct injection of modified WT1 peptide as a vaccine can induce a WT1-specific immune response [253]. OHNO et al. [254] performed a phase II clinical trial investigating a WT1 vaccine in patients with WT1/HLA-A 2402-positive gynecological cancers. Twelve enrolled patients were given 3.0 mg of HLA-A 2402-restricted, modified 9-mer WT1 peptide every week for 12 consecutive weeks. During the 3 months trial period, the disease control rate was 25.0%; however, no cases experienced a CR or PR with this therapy. To explore a more effective vaccination, administration of autologous DCs loaded with WT1 peptides gained attention. In 2013, Coosemans et al. [253] published a report of a Phase I/II Trial (EudraCT 2009-016868-37) investigating WT1-loaded dendritic cell immunotherapy in patients with uterine tumors. Among the 6 enrolled patients, 3 were diagnosed with serous endometrial carcinoma. Four of the patients were HLA-A2-positive including 2 EC patients. All patients were given autologous DCs transfected with WT1 mRNA as vaccines weekly for 4 weeks, and 2 HLA-A2-positive patients received 2-3 additional injections. As a result, 75% (3/4) of HLA-A2-positive patients showed an immunological response after four injections including the 2 HLA-A2-positive EC patients who also demonstrated increased WT1-specific T-cells and NK cells. No oncological or immunological response was observed in the 2 HLA-A2-negative patients which included 1 EC patient.

As granulocyte-macrophage colony-stimulating factor (GM-CSF) has been found as the most valid anti-tumor cytokine through systematic selection on cytokine panels in murine models [255], combining cancer vaccines with GM-CSF might have a synergistic anti-tumor effect. Folate-binding protein is highly expressed in various malignant tumors [256, 257]. E39 is an immunogenic, HLA-A2-restricted peptide derived from folate-binding protein and is the most consistent recognition site of tumor-associated lymphocytes. In a phase I/IIa trial (NCT01580696) [258], the clinical activity of E39 peptide vaccine in combination with GM-CSF was explored in ovarian and endometrial cancer patients. Fifty-one enrolled patients were divided into 2 groups: 1. vaccine group: HLA-A2 positive cases receiving E39+GM-CSF vaccines (29 cases) and 2. control group: HLA-A2 negative cases or HLA-A2 positive cases rejecting vaccines (22 cases). Nine EC patients were enrolled in this trial: 6 in the vaccine group and 3 in the control group. The 24-month DFS was 55.5% for all vaccine group patients and 40.0% for all control group patients (P= 0.339). The 24-month DFS of EC patients in vaccine group was 62.5%, which was higher than that of total vaccine group. The vaccine was well tolerated with no greater than G2 local toxicities and no greater than G3 systemic toxicities observed.

NY-ESO-1 is a cancer/testis antigen which expressed both on testis and various human malignancies [259–261]. Jäger et al. [262] conducted a trial to evaluate the safety and immunogenicity of using NY-ESO-1 vaccine in patients with 8 kinds of advanced NY-ESO-1-expressing tumors. Thirty-six enrolled patients were given recombinant vaccinia-NY-ESO-1 (rV-NY-ESO-1) and a fowlpox-based vaccine containing NY-ESO-1. Twenty-three patients finished four vaccinations and entered the final analysis for tumor and immunological response. NY-ESO-1-specific T cell and antibody responses were observed in the majority of cases. The different patterns of immune responses observed in patients after treatment were divided into four categories, from the lowest category I to the highest category IV, based on serologic conversion and T cell response. The single stage IV EC patient was classified into Category III, which included converting from sero-negative to sero-positive and improved CD4+ and/or CD8+ T cell responses. Although she showed obvious immunological response, she had progressive disease.

In addition to tumor associated antigens, epitopes of immune cells are good candidates for manipulating vaccines. A phase I trial in patients with metastatic malignancies published in 2009 by Kaumaya et al. [263] evaluated a chimeric peptide vaccine which combined B-cell epitopes derived from the human epidermal growth factor receptor 2 (HER2) extracellular domain with a promiscuous T cell epitope. This combination vaccine was emulsified in Mon-tanide ISA 720 (SEPPIC, Inc., Paris, France) with nor-muramyl-dipeptide (n-MDP) adjuvant. Twenty-four patients received 3 vaccinations and 25% (6/24) exhibited clinical benefit. Two EC patients were included in the final analysis with only 1 having a clinical PR. This study demonstrated that the chimeric vaccine was safe as no greater than G3 treatment related AES were observed. This type of combination vaccine was further studied, and a recent publication in 2019 of a phase I trial (NCT01417546) showed antitumor activity with this vaccine type in patients with advanced solid tumors in a phase I trial, but no EC patients were included [264].

#### Nucleic acid-based vaccines

mRNA-4157 is a personalized vaccine designed by a proprietary algorithm. It is a lipid encapsulated personalized vaccine which elicits specific anti-tumor T cell responses through the vaccine encoding neoantigens. A phase 1 Keynote-603 study (NCT03313778) is ongoing to evaluate the safety and immunogenicity of mRNA-4157 monotherapy in patients with resected solid tumors or mRNA-4157 plus pembrolizumab in patients with unresectable solid tumors. In the monotherapy group, mRNA-4157 was given 0.04 - 1 mg every 3 weeks for 9 cycles; in the combination group, patients first received 2 cycles of 200 mg pembrolizumab, then received mRNA-4157 plus pembrolizumab for 9 cycles, which may be followed by a pembrolizumab monotherapy for up to 2 years. In May 2019, an interim report of this trial was published and up to 33 patients were enrolled in the treatment. One MSI-high EC patient was included in the combination therapy cohort, with the remaining tumor types including bladder , HNSCC, melanoma, NSCLC, SCLC, MSI-high CRC, MSI-high prostate and TMB-high metastatic cutaneous squamous cell. Although the outcome of the only EC patient was not provided, the clinical responses for the whole combination therapy cohort, including 5 PRs, 6 SD, and 8 PD, were noted with no SAEs or AEs ≥ G3 supporting the advancement of mRNA-4157 to future trials.

In MSI EC cells, MMR deficiency can result in length changes of microsatellite sequences within coding regions of the human genome, which is termed as coding MSI [265]. Coding MSI can generate frameshift peptides (FSPs) which can promote oncogenesis through functional inactivation of tumor-suppressive proteins and induce tumor-specific immune responses [266]. FSPs are not found on normal human cells, and the expression patterns of MSI-related FSPs between sporadic MSI tumors and hereditary MSI tumors show no difference. Thus, based on the immunogenicity and consistent expression in MSI tumors, MSI-related FSPs have become candidates for developing cancer vaccines in MSI EC. In 2019, a phase I, First In Humans, multicenter study of Nous-209 genetic polyvalent vaccine combined with pembrolizumab in patients with MSI solid tumors was posted and is currently recruiting patients. Nous-209 Genetic Vaccine is a heterologous prime/boost regimen composed of GAd20-209-FSP (priming) and MVA-209-FSP (boosting), which encodes 209 distinct FSP cancer neoantigens found in various MSI tumors including MSI EC. Thirty-four patients are estimated to be enrolled and will be administrated 1 priming dose of GAd20-209-FSP followed by 3 boots with MVA-209-FSP in combination with pembrolizumab. Dose limited toxicity and Treatment-Emergent AEs will be evaluated for this regimen as primary outcome measures.

### ACT

ACT, a passive immunotherapy, means isolating allogenic or autologous immune cells, which are activated and expanded ex vivo followed by reinfusion into cancer patients [267]. This form of immunotherapy has several advantages, including production of multiple tumor-specific lymphocytes in vitro, alternative engineered immune cells for specific tumor antigens and favorable host environment due to lymphodepletion prior to ACT [268]. CTL and NK cell are principal effector cell types used in ACT, while DC are usually used as a tool to carry vaccine or to present antigens to stimulate T cells in vitro [268, 269]. Additionally, transforming genetically modified T or NK cells (such as CAR T or CAR NK cells) are emerging strategies in ACT and have shown activity in a majority of malignancies. However, ACT also has several limitations: short-period effects and poor trafficking of effective cells within immunosuppressive environment [268]. Current studies related to ACT in EC involve transferring Lymphokine-activated killer (LAK) cells, common DC primed T cells, CAR T cell therapy and other complex combination therapies.

### Adoptive LAK cells transfer

LAK cells is a group of lymphocytes mainly comprised of T and NK cells which are generated by patient derived peripheral blood mononuclear cells exposed to high dose interleukin-2 (IL-2) [270–272]. The preclinical study of LAK cells in EC was first published in 1989. Shimizu et al. [273] proposed that the combination of adoptive transfer of LAK cells in combination with intraperitoneal injection of recombinant IL-2 (rIL-2) markedly inhibited the growth of human EC cell line xenografts in nude mice. The adoptive transfer of LAK cells plus IL-2 was further performed in clinical patients in a phase I trial by Steis et al. [274]. Twelve patients with colorectal cancer, 10 patients with OC, 1 patient with small-bowel adenocarcinoma, and 1patient with EC, all with malignancies limited to the peritoneal space, were enrolled in this trial. A modest therapeutic efficacy was observed as 30% of the patients had PR in this trial. However, the single one EC patient showed no responses. Additionally, this kind of ACT requires improvement as multiple and significant toxicity-related AEs were observed. All patients involved developed diffuse abdominal pain and rebound abdominal tenderness, which was assumed to be partly related to the injection of LAK cells. Treatment related intraperitoneal fibrosis was another severe complication of this strategy.

#### Adoptive T cell transfer

Adoptive T cell transfer is a significant component of ACT therapy. Santin et al. [275] investigated the adoptive transformation of DC primed peripheral blood T cells in a 65-year-old woman with unresectable and chemoresistant EC. The autologous DC were first treated with autologous tumor lysate and then were used to generate activated tumor-specific T cells. During the treatment, the patient showed stabilization of a liver metastasis which had markedly increased from 9.5 x 8 cm to 14 x 10 cm in the 3 weeks prior to the treatment. The patient showed a SD response during the treatment and maintained a stable status for at least 3 weeks after the final infusion.

T cells can be genetically modified through introducing chimeric antigen receptors (CAR) by retrovirus or a lentivirus [276]. CARs are comprised of 3 modules: an extracellular target binding module, a transmembrane module, and an intracellular module [277], which can provide both tumor-associated antigens recognition and T cell activation [278]. CAR T-cell therapy was a breakthrough in the treatment of lymphoid malignancies, such as Acute Lymphoblastic Leukemia (ALL) [279, 280] and Diffuse Large B-cell Lymphoma (DLBCL) [281]. CAR T-cell therapy related research results are still lagging behind in EC. Rodriguez-Garcia et al. [282] established a preclinical study of anti-Müllerian inhibiting substance type II receptor (MISIIR) CAR T cell therapy in patients with ovarian and endometrial cancer. The MISIIR, as a member of the transforming growth factor-β receptor family, is highly expressed on the majority of gynecologic cancers [283–288], making it an ideal target for CAR T cell therapy. The anti-tumor activity of anti-MISIIR CAR T cell therapy in EC was examined by a study of in vitro co-culture CAR T cells with AN3CA (a human EC cell line) and in vivo AN3CA xenograft mice models. At the end of the treatment, when compared with 2 control groups, the experimental group which used MISIIR-specific CAR T cell therapy showed higher levels of IFN-γ in supernatants, approximately 2-fold shrinkage of tumor volume, higher concentrations of circulating CD3+T cells, and higher percentages of CD45+cells infiltrating in tumors.

#### Combination of adoptive T cell transfer with ICBs

Combining ACT with ICBs or traditional therapeutic methods may increase the potency of targeting and eliminating EC. In a phase I study (NCT03757858), Qiao et al. investigated several combination strategies of autologous ACT with other therapeutic methods in patients with advanced solid tumors. Thirty-three patients were divided into 3 therapeutic cohorts: 10 patients receiving hyperthermia plus ACT, 11 patients receiving hyperthermia plus ACT plus pembrolizumab and 12 patients receiving hyperthermia plus ACT plus chemotherapy. Hyperthermia can exert antitumor activity and have a synergistic effect with chemotherapy as a thermal sensitizer [289]. One EC patient was in the hyperthermia plus ACT cohort while both the hyperthermia plus ACT plus pembrolizumab and hyperthermia plus ACT plus chemotherapy cohort included 2 EC patients. Although the total ORR was 30% (10/33) and 3 patients achieved CR, the ORR for 5 EC patients was 20%, and no EC patient had a CR (1 PR, 2 SD and 2 PD for EC cohort). There was no immune related AEs in this trial with toxicities caused by chemotherapy and hyperthermia and mostly identified as G1-2(13/15 patients, 86.7%).

### Immunotherapy in p53 mutant subtype

As mentioned earlier, the TCGA molecular classification [14] initially defined the molecular characteristics of the p53 mutant/serous-like subtype of EC and verified the genetic similarity of this type of EC with HGSOC, and basal-like breast carcinomas (BLBC) (account for 55-81% of triple-negative breast cancer, TNBC) [290, 291]. There are shared amplification mutations among the serous EC, HGSOC and BLBC such as *MYC, ERBB3, CCNE1, MCL1, MECOM* and *FGF3* [14]. Additionally, serous EC patients have a higher frequency of *ERBB2* amplification compared with those with HGSOC and BLBC, and most of the *ERBB2* amplified EC cases have concurrent *PIK3CA* mutations [14]. Based on precision medicine, these molecular characteristics can guide clinical treatment including immunotherapy. It is increasingly appreciated that emerging treatment of HGSOC and TNBC can provide lessons for *TP53* mutant EC, and separate consideration of novel strategies may benefit this EC subgroup [292].

For HGSOC [293] and TNBC [294], no official immunotherapy has been approved, and efforts are currently concentrated on the development of combination treatment of immunotherapy with other strategies. The phenotype of BRCAness is tightly associated with basal-like sporadic breast tumors and TNBC [295], and homologous recombination deficiency is observed in more than 50% of HGSOC patients [296]. Abnormal cell cycle related genes and the high frequency of *TP53* mutations in copy-number high EC suggest vulnerability to DNA damage and repair dysfunction [292]. PARP inhibitors have shown great clinical activity in DNA repair deficient carcinomas. Recent experimental studies found that upregulation of PD-L1 induced by PARP inhibitors can produce immuno-tolerance during the cancer treatment, which can be blocked by PD1/PD-L1 antibodies. The TOPACIO (Keynote 162) trial (NCT02657889) is ongoing and is investigate the treatment of Niraparib (a type of PARP inhibitor) with pembrolizumab in patients with metastatic TNBC or OC. The data suggest adding PARP inhibitors to immunotherapy as a combination strategy may be potentially beneficial for copy-number high EC. Moreover, the mutation of *PIK3CA* gene, which encodes the PI3K catalytic subunit α and plays a role in PI3K/AKT/mTOR pathway, is frequently found in TNBC (10.2%) [295] and copy-number high EC [14]. The amplification of *MYC*, which can be targeted by MEK inhibitors, is also observed in a variety of serous EC, HGSOC [14], and 30% of TNBC or BLBC patients [297, 298]. A phase Ib trial (NCT02900664) investigating the treatment of advanced adenocarcinoma, including TNBC, with PD-L1 antibody plus MEK inhibitor is currently ongoing. Treatment-emergent AEs and dose limiting toxicities (DLTs) will be evaluated. These data suggest adding specific molecular targeted agents, such as PI3K/AKT/mTOR pathway and MEK inhibitors, to immunotherapy may provide powerful anti-tumor effects in copy-number high EC.

Recent studies have demonstrated that IDO is a critical molecular in inducing immuno-tolerance in HGSOC and TNBC. IDO1 positivity is found in 37% of all TNBCs and has a tight association with basal-like TNBC [299]. The African American Cancer Epidemiology Study (AACES) [300] demonstrated positive IDO expression in 58% of HGSOC, and most PD-L1-positive patients co-expressed IDO. Both of markers were associated with higher lymphocyte infiltration (P<0.05). Epacadostat (epac), an oral IDO inhibitor, can restore or promote the proliferation of dendritic cells, NK cells, and effector T cells as well as decreasing Treg cells. A combination treatment of epacadostat plus pembolizumab is being tested in a phase I/II, Keynote 037-ECHO 202 study (NCT 02178722) in patients with selected cancers including TNBC. Although there are limited data about the expression status and related treatment of IDO in copy-number high EC, it is important to explore the IDO related immunotherapy in this subgroup.

Amplification of ERBB2 (17q12) is found in 26-62% of uterine serous cancer patients [301–304] and has been defined as one of the significant amplified oncogenes in copy-number high EC [14]. Thus, human epidermal growth factor receptor 2 (HER2/Neu, also known as ERBB2) targeted immunotherapy has been considered as a powerful treatment for this subtype of EC. This kind of treatment employs humanized monoclonal antibodies targeting HER2, such as trastuzumab and pertuzumab, to recruit NK cells via Fc region conjugation [305] and kill the tumor cells through antibody-dependent cell-mediated cytotoxicity (ADCC) and complement-dependent cytotoxicity (CDC) effects [306–308]. However, in a GOG-181B trial [309] investigating the efficacy of trastuzumab monotherapy in patients with stage III/IV, recurrent, HER2-positive EC, trastuzumab showed no clinical activity in EC with HER2 overexpression or amplification. However, only 28% (7/25) of serous carcinoma patients were HER2 positive and able to be enrolled in this trial. Recently, Fader et al. [310] published the results analysis of a multicenter, randomized phase II trial of treating HER2 overexpressed advanced serous EC with carboplatin-paclitaxel (control) or carboplatin-paclitaxel plus trastuzumab (experimental). Fifty-eight patients were evaluated, and the median PFS in the control group vs experimental group was 8.0 vs 12.6 months (P=0.005; hazard ratio [HR], 0.44; 90% CI, 0.26 to 0.76), suggesting improved outcomes with the addition of trastuzumab to basic chemotherapy. Toxicity was not different between the control and experimental groups.

BiTE antibody (bispecific antibody) is a novel drug which bridges cancer cells with cytotoxic T cells and induces a direct cytolytic effect of T cells without the restriction of specific T cell receptor, MHC class I molecules, or peptide antigen presentation [311–313]. Bellone et al [314] performed a preclinical study using BiTE antibodies (solitomab) in patients with epithelial-cell-adhesion-molecule (EpCAM) over-expressed uterine serous EC(USC). EpCAM expression is found in 87.5% of USC which has been demonstrated to be susceptible to solitomab, a type of EpCAM/CD3 bispecific antibody. The clinical efficacy of solitomab has been noted in multiple cancers such as colon cancer and OC [315, 316]. In this preclinical study of EC, the solitomab treated group displayed increased cytokine secretion, T lymphocyte activation and proliferation, as well as cytotoxic activity of tumor associated lymphocytes, suggesting clinical value of this drug [314].

### MetS & immunotherapy

MetS represents a group of risk factors related to the development of diabetes and cardiovascular disease [317]. Obesity, diabetes and hypertension, as the common phenotype of MetS, are often found as a metabolic triad in EC patients [318]. The risk of EC in overweight (BMI≥ 25 kg/m2), diabetic, or hypertensive patients were, respectively, 2.45, 2.12, and 3.5 times higher than control groups [318]. Based on a series of retrospective and prospective studies, EC is now defined as one of the tumor types most closely associated with MetS [1]. MetS can promote EC development through complex modulating mechanisms, local inflammation [319], and remodeled immune microenvironment [318, 320].

Obesity is one of the key characteristics of MetS and adipose tissue plays an essential role in the pathophysiological changes and tumor-microenvironment interaction in EC [320]. In obese people, the balance between adipocytes and immune cells is impaired. Obesity-related chronic adipose inflammation gradually develops [321] and promotes tumor progression [322, 323]. A cluster of pro-inflammatory cytokines, such as IL-6, TNF-α and IL-18, are secreted by adipocytes to facilitate the infiltration of lymphocytes in EC cells [324], resulting in angiogenesis [325], tissue remodeling [319], and a pro-neoplastic microenvironment [321]. The local inflammatory mechanism promotes malignant transformation of normal tissues and tumor development [326].

Systemic hyperglycemia is a characteristic of diabetes which serves as a favorable metabolic environment for the rapid proliferation of cancer cells [318]. In obese EC patients, cancer cells produce high levels of lactic acid (a metabolite of glycolysis) during hyperglycemia, which can promote malignant transformation of normal cells through metabolism re-programming [318] and also transform anti-tumor M1 macrophages to pro-tumor M2 macrophages [327]. Insulin resistance coupled with hyperinsulinemia and up-regulated insulin growth factor-1 are not only seen in diabetes but also has a tight association with hyperglycemia, obesity, and cancer development [328].In MetS patients, obesity-related inflammation induces the expression of a cluster of inflammatory cytokines including C-reactive protein (CRP), IL-6, TGF-α, and plasminogen activator inhibitor-1 (PAI-1) [329, 330], which further influence insulin signaling pathways and lead to insulin resistance. Elevated insulin and IGF-1 can combine with IR and IGF-1 receptors (IGF-1R) respectively and promote EC cell proliferation via downstream signal transformation [331]. Metformin is a type of insulin-sensitizing anti-hyperglycemic drug which is widely used in treating type II diabetes mellitus [332, 333]. Several new applications for this agent have recently been found, one of which is its anti-tumor effects [334]. Metformin has shown promising efficacy as a new adjunctive treatment in EC. A systematic review and meta-analysis raised by Meireles et al. [335] demonstrated that metformin treatment led to reversion of atypical endometrial hyperplasia to normal endometrium with down-regulated proliferation markers (from 51.94% to 34.47%, CI = 36.23-67.46% and 18.55-52.43% ). Higher OS was also observed in metformin-treated EC patients compared with non-metformin-treated and non-diabetic patients (HR =0.82; CI: 0.70-0.95; P=0.09, I [2]=40% ). Prior studies have identified several anti-tumor mechanisms of metformin [336–338] with adenosine monophosphate-activated protein kinase (AMPK)-dependent PI3K/Akt/mTOR pathway inhibition considered as an important direct mechanism of metformin in treating EC [333, 339]. Furthermore, metformin also has immune-mediated antitumor effect including blocking the PD-L1/PD-1 axis [340], increasing CD8+TIL infiltration, and protecting CD8+TILs from apoptosis and exhaustion [341]. Based on its immune-related effects, preclinical studies have found synergistic antitumor effects by combining metformin with cancer vaccines [341] and CTLA4 blockades [340]. Though there is no clinical study testing metformin combination immunotherapy, it is promising to explore the efficacy of this combination strategy.

### Hormone & immunotherapy

Hormone aberrations, such as estrogenic excess, lack of progesterone and abnormal expression of endometrial receptors, have long been considered as significant etiological factors for EC [342, 343]. Elevated estrogen results in angiogenesis, endometrial cell proliferation and apoptosis inhibition [344], thus promoting carcinogenesis. Progesterone deficiency decrease its protective effects on endometrial epithelial cells from malignant transformation, such as inducing cells apoptosis via binding to PR [345], inhibiting ERα expression, and controlling growth factor production of stromal cells [346, 347]. Sex hormones also have immuno-modulatory functions which participate in tumor-immunity interactions. Almost all types of immune cells express receptors for progesterone and estrogen [348–351], and ER and androgen (AR) responsive elements have been found located on the promoters of many immune-related genes [352]. In fact, one sex hormone can have both immune stimulatory and immune inhibitory functions based on the doses and time of action [353]. Estrogen can both promote inflammation through inducing IFN-γ and IL-2 secretion by peripheral T cells [354] and also induce immune tolerance via stimulating IL-10 production [355]. Progesterone can enhance humoral immune responses and the production of IL-5, IL-6, and IL-10 [356], while also inhibiting T cell proliferation [357] and IFN-γ production [358]. Witkiewicz et al [359] investigated the use of progestin in 15 patients with complex atypical endometrial hyperplasia and well-differentiated EC who desired fertility preservation or were unsuitable for surgery. After completion of the treatment, 66.7% (10/15) of patients had normal morphology on follow-up sampling while 26.7% (4/15) of patients had persistent or progressive disease. Progestin was found to significantly influence the subpopulations of lymphocytes as decreased Tregs and increased NK cells were observed in post-treatment specimens. Because of this complex immune-regulatory mechanism about which research has been limited the roles of sex hormones in immuno-oncology still remain to be identified in EC.

In EC patients, deficiency of both ER and PR expression is an independent prognostic marker for worse outcomes, even in those defined as low-grade [360]. The double negative of ER and PR is also considered a predictor for lymph node metastases [361] and tumor relapse [362]. Furthermore, single deficiency of PR expression predicts poor outcome in high-grade EC patients, even in those whose histotypes, such as serous type, were previously recognized as hormone-independent types [363]. The ER-positive rates are similar among the four TCGA molecular subtypes (POLE:75.7%, MSI:73.9%, *TP5* WT:92%, *TP53* mutant:67.4%), while the PR-positive rate in *TP53* WT group is the highest (POLE: 75%, MSI: 60.9, *TP53* WT: 83.9%, *TP53* mutant: 44.7%) [364]. An analysis of early stage EC in the PORTEC cohorts showed a higher ER/PR-negative rate in *TP53* mutant group [365]. These data provide basis for stratifying clinical risks within different molecular phenotypes. Recent studies have proposed controversial opinions on the relationship between ER and PR expression status and immune responses in EC. Jiang et al. [366] found that the infiltration of tumor-associated macrophages was higher in PR-negative EC cases compared with positive ones (P = 0.0001), which predicted immuno-tolerance of tumors and poor outcomes. However, Giatromanolaki et al. [367] suggested that a low infiltration of FOXP3+ Treg cells, which participate in pro-tumor immune responses, was associated with ER-negative and low vascular density in EC.

Ongoing trials of endocrine therapy are fewer than those of chemotherapy and new target therapies [368]; however, endocrine therapy still warrants attention due to its good tolerance and known toxicity [368]. The combination of endocrine therapy with immunotherapy may be an alternative option for young patients seeking fertility sparing therapies. A phase 1 trial (NCT04046185) is now ongoing to investigate the combination of Toripalimab with progesterone in Stage I, FIGO grade 1-2 endometrioid EC patients who desire fertility preservation. The pathologic complete/partial remission rates will be evaluated at 6 months after the treatment initiation.

## Conclusion

In 1893, William Coley, a New York orthopedic surgeon, accidentally observed that the tumor tissue of patients infected with Streptococcus pyogenes could make the tumor tissue of some patients slowly recede, which opened the prelude of tumor immunotherapy [369]. Anti-tumor immunotherapy has achieved ideal results in the treatment of malignant tumors, but there are still many problems in clinical application that have not been resolved. The reason is that the understanding of tumor immune mechanism is not thorough enough currently, and more research is needed to reveal new cellular and molecular mechanisms. For the problems that arise in immunotherapy, it is first necessary to formulate individualized treatment strategies to achieve individualized precision treatment. Secondly, it is necessary to establish a set of reasonable evaluation standards reflecting the effect of immunotherapy. Finally, it is necessary to overcome tumor immune tolerance. While using the activated immune system to treat tumors, it is necessary to reduce the immunosuppressive environment in patients, especially the immunosuppressive microenvironment inside tumor tissues. With the accumulation of more clinical experience and the development of scientific research, we will have a deeper understanding of tumor immunotherapy, and we have reason to believe that this novel treatment method can be applied to cancer patients more safely and effectively.

## Data Availability

Not applicable.

## Abbreviations

ICIs: Immune checkpoint inhibitors
ACT: Adoptive cell transfer
EC: Endometrial cancer
MetS: Metabolic syndrome
ICBs: Immune checkpoint blockades
RT: Radiotherapy
POLE: Polymerase-ε
MSI-H: Microsatellite instability hypermutated
MMR: Mismatch repair
MSS: Microsatellite stability
HGSOC: High-grade serous ovarian carcinoma
NK: Natural killer
Treg: Regulatory T cells
DCs: Dendritic cells
CTLs: CD8+ cytotoxic T cells
IDO: Indoleamine 2, 3-dioxygenase
MDSC: Myeloid derived suppressor cells
APC: Antigen-presenting cell
PD1: Programmed cell death 1
CTLA4: Cytotoxic T lymphocyte antigen 4
PDL1: Programmed cell death 1 ligand 1
NSCLC: Non-small-cell lung cancer
FDA: Food and drug administration
OS: Overall survival
SCLC: Small-cell lung cancer
ORR: Objective response rate
TILs: Tumor-infiltrating lymphocytes
OC: Ovarian cancer
DFS: Disease free survival
LS: Lynch syndrome
COX-2: Cyclooxygenase-2
IFN-γ: Interferon-γ
ARID1: AT-rich interaction domain 1A
CR: Complete response
SD: Stable disease
AEs: Adverse events
PD: Progressive disease
rEC: Recurrent endometrial cancer
IC: immune cells
DOR: Duration of objective response
OR: Objective response
PFS6: PFS at 6 months
OTR: Objective tumor response
AEC: Advanced endometrial cancer
PARP: Poly (ADP-ribose) polymerase
ADCs: Antibody-drug conjugate
LAG-3: Lymphocyte-activation gene 3
MTD: Maximum tolerated dose
RD: Recommended dose
TIM-3: T cell immunoglobulin and mucin-domain containing-3
GM-CSF: Granulocyte-macrophage colony-stimulating factor
HER2: Human epidermal growth factor receptor 2
FSPs: Frameshift peptides
LAK: Lymphokine-activated killer
IL-2: Interleukin-2
rIL-2: Recombinant IL-2
CAR: Chimeric antigen receptors
MISIIR: Müllerian inhibiting substance type II receptor
BLBC: Basal-like breast carcinomas
TNBC: Triple-negative breast cancer
DLTs: Dose limiting toxicities
EpCAM: Epithelial-cell-adhesion-molecule
BC: Breast cancer
CC: Cervical cancer
